# Multiple Xanthogranulomas in an Adult Patient with Myelodysplastic Syndrome

**DOI:** 10.1155/2020/8826715

**Published:** 2020-12-07

**Authors:** Marta Martínez-García, Nicolás Silvestre-Torner, Antonio Aguilar-Martínez, Fernando Burgos-Lázaro

**Affiliations:** ^1^Dermatology Service, Severo Ochoa University Hospital, Leganés, Madrid, Spain; ^2^Pathological Anatomy Service, Severo Ochoa University Hospital, Leganés, Madrid, Spain

## Abstract

Adult multiple xanthogranuloma (XG) is a rare late-onset variant of juvenile XG. It is characterized by the appearance of papules or nodules located preferably on the trunk. A case of a 54-year-old man with myelodysplastic syndrome is presented as a history of interest, who consulted due to the appearance of multiple brownish papules distributed mainly in the trunk. So far, there are only 22 cases of this clinical form reported in the literature, 9 of them associated with malignant hematological processes. We highlight the importance of this entity as a possible cutaneous marker of blood dyscrasias.

## 1. Introduction

Xanthogranuloma (XG) is the most common form of clinical presentation of non-Langerhans cell histiocytosis. Although it can appear at any time of life, XG usually occurs in pediatric age and generally as a single lesion. The appearance of XG in adult patients is very rare and usually occurs in the form of multiple lesions. The association of XG with different blood dyscrasias has been documented both in the juvenile form and in the adult form, although in the latter in a more isolated way.

## 2. Case Presentation

A 54-year-old male came to the consultation with a 1-year history of clinical history indicating the appearance, spontaneously, of multiple skin lesions distributed on the trunk. The lesions showed a progressive increase in number and size and were not associated with local symptoms. In the personal history, the presence of a myelodysplastic syndrome with low-risk unilinear IPSS-R dysplasia, with an IDH 1 mutation, diagnosed 3 years ago for which he maintained periodic control without treatment, stood out.

**Figure 1:**
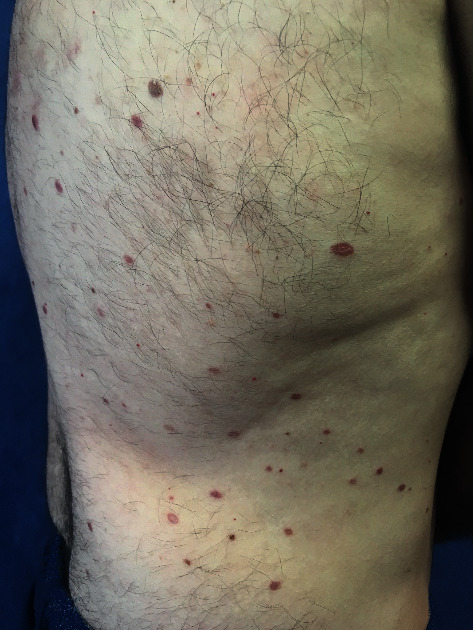
Multiple yellow-brown papules in the trunk.

**Figure 2:**
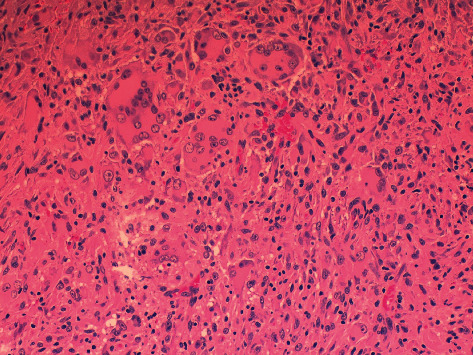
Mixed proliferation with elongated cells and multinucleated histiocytic-like cells of large eosinophilic cytoplasms with mild lymphocyte response (H-E ×20).

**Figure 3:**
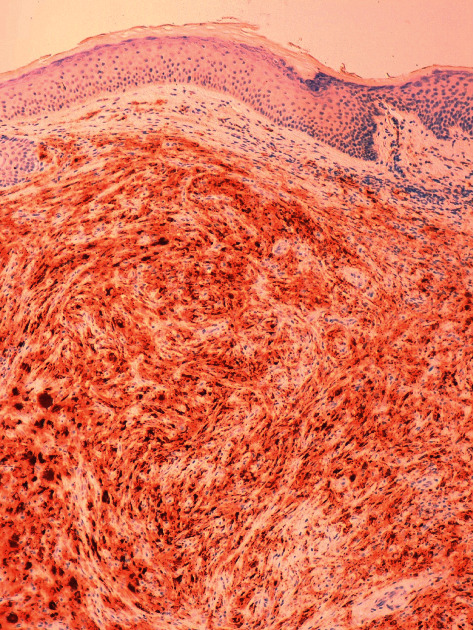
Positivity for histiocytic markers CD 68 (×10).

**Figure 4:**
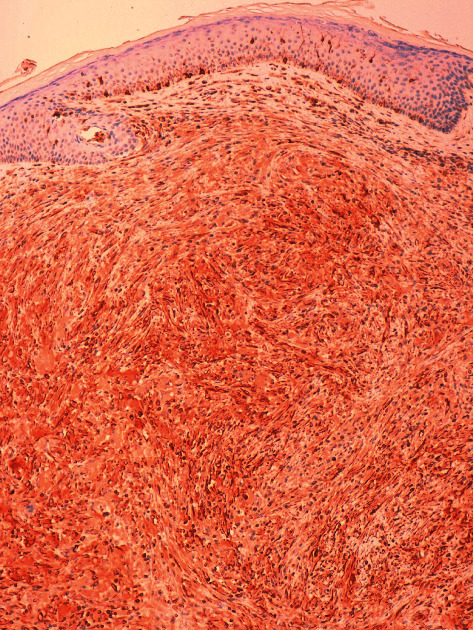
Positivity for vimentin (×10).

**Figure 5:**
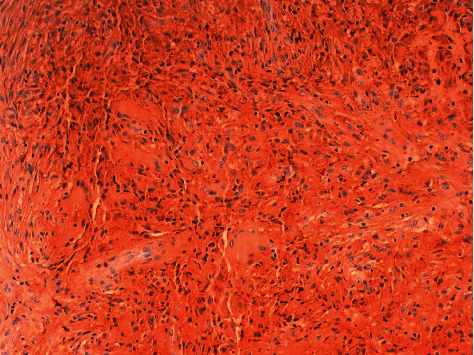
Positivity for alpha1 antitrypsin (×20).

A dermatological examination revealed multiple papules between 3 and 5 millimeters in size, yellow-brown in color, distributed mainly in the trunk (Figure 1). The rest of the skin examination showed no data of interest. A histological study was performed that showed dermal occupation by mixed proliferation with elongated cells and multinucleated histiocytic-like cells of large eosinophilic cytoplasms with mild lymphocyte response. No significant atypia, mitosis, or necrosis was observed (Figure 2). The immunohistochemical study showed positivity for histiocytic markers CD 68 (Figure 3), vimentin (Figure 4), and alpha1 antitrypsin (Figure 5) and negativity for S-100, CD1a, factor XIIIa, and cytokeratins.

The hematological study carried out included bone marrow cytology with discrete hypocellularity with dysplasia in 39% of the granulocytic series. Nucleus-cytoplasmic maturation asynchrony was observed in the red series. No increase in the number of blasts was observed. In bone marrow cytometry, hypogranularity was observed in the granulocytic series without excess blasts. The following IDH1, TET2, and KMT2A mutations were found in the NGS molecular biology study.

The patient maintains periodic dermatological and hematological control. The skin lesions remained stable, and no type of treatment has been performed.

## 3. Discussion

XG usually appears in children, mainly in the first year of life, although it can appear at any time of life. The usual form of presentation is a single papule or nodule usually located on the head or neck. The appearance of XG in adults is infrequent, with only 22 cases described in the literature [1–8], being the usual presentation in these cases the multiple forms (10 or more lesions) that are preferably located on the trunk.

Histologically, the findings are similar in both the juvenile and adult forms [9]. A dense foam cell, giant cell, Touton cell, lymphocyte, and eosinophil infiltrate located at the level of the reticular dermis with extension to subcutaneous cell tissue are observed. Immunohistochemistry is positive for macrophage markers such as CD68, HAM56, and factor XIIIa. Negativity for CD 1a and S-100 allows them to be differentiated from Langerhans cell histiocytosis [10–12].

In most patients, XG lesions are located exclusively at the cutaneous level, but the presence of extracutaneous lesions have been described in both juvenile and adult forms [13], with the eyeball being the most frequently affected organ. The association between juvenile XG, neurofibromatosis type 1, and chronic myeloid juvenile leukemia (CMJL) has been documented with approximately 20 patients reported in the literature [14]. A risk of developing CMJL of 0.4%–1.6% has been estimated in children with neurofibromatosis type 1 and juvenile XG [15, 16].

In adult onset forms, association with blood dyscrasias has been described in a total of 9 patients and includes follicular lymphoma, essential thrombocytosis, chronic lymphatic leukemia B, acute lymphatic leukemia B, large-cell lymphoma b, monoclonal gammopathy, adult T lymphoma/leukemia, and a single patient with myelodysplastic syndrome [3, 4, 6–8, 17, 18]. A case associated with a solid organ tumor has also been documented [19]. We must emphasize that all the patients presented multiple XG and that the development of the cutaneous lesions took place before, during, or after the hematological process. The etiopathogenic mechanism that would justify this association is unknown. Anomalous proliferation of histiocytes from a bone marrow CD 34+ precursor that would be activated against different stimuli such as the induction of cytokines or gammaglobulins produced by the underlying tumor process has been suggested [4, 6, 20–22].

The evolution is usually towards spontaneous regression, less frequently in adults and in multiple forms [12]. In cases associated with blood dyscrasias, a tendency to regression has been observed when treating neoplasia [19]. There is no specific treatment for XG. In most cases, an expectant attitude is recommended, although CO_2_ laser, simple excision, and cryotherapy have been used.

Due to the low number of published cases, there is no agreement on the management of XG in adults, although it seems advisable to perform a physical examination together with a periodic hematological study.

In conclusion, we highlight the importance of recognizing these forms of multiple XG in adults as a possible marker of blood dyscrasias, describing a case of multiple XG in an adult patient associated with myelodysplastic syndrome.

## Data Availability

No data were used to support this study.
